# Regulation of Copper Metabolism by Nitrogen Utilization in *Saccharomyces cerevisiae*

**DOI:** 10.3390/jof7090756

**Published:** 2021-09-14

**Authors:** Suzie Kang, Hyewon Seo, Min-Gyu Lee, Cheol-Won Yun

**Affiliations:** School of Life Sciences and Biotechnology, Korea University, Anam-dong, Sungbuk-gu, Seoul 02841, Korea; sthe327@korea.ac.kr (S.K.); hyewon330@korea.ac.kr (H.S.); leemg@korea.ac.kr (M.-G.L.)

**Keywords:** *S. cerevisiae*, copper, iron, nitrogen, Mac1

## Abstract

To understand the relationship between carbon or nitrogen utilization and iron homeostasis, we performed an iron uptake assay with several deletion mutants with partial defects in carbon or nitrogen metabolism. Among them, some deletion mutants defective in carbon metabolism partially and the *MEP2* deletion mutant showed lower iron uptake activity than the wild type. Mep2 is known as a high-affinity ammonia transporter in *Saccharomyces cerevisiae*. Interestingly, we found that nitrogen starvation resulted in lower iron uptake activity than that of wild-type cells without downregulation of the genes involved in the high-affinity iron uptake system *FET3/FTR1*. However, the gene expression of *FRE1* and *CTR1* was downregulated by nitrogen starvation. The protein level of Ctr1 was also decreased by nitrogen starvation, and addition of copper to the nitrogen starvation medium partially restored iron uptake activity. However, the expression of *MAC1,* which is a copper-responsive transcriptional activator, was not downregulated by nitrogen starvation at the transcriptional level but was highly downregulated at the translational level. Mac1 was downregulated dramatically under nitrogen starvation, and treatment with MG132, which is an inhibitor of proteasome-dependent protein degradation, partially attenuated the downregulation of Mac1. Taken together, these results suggest that nitrogen starvation downregulates the high-affinity iron uptake system by degrading Mac1 in a proteasome-dependent manner and eventually downregulates copper metabolism.

## 1. Introduction

The budding yeast *Saccharomyces cerevisiae* has been used as a model system to investigate diverse nutrient metabolism pathways, and many studies on metal metabolism have been performed [1,2,3,4]. In *S. cerevisiae*, iron metabolism is regulated strictly because iron can have toxic effects on cells by forming hydroxyl radicals via the Fenton reaction [5]. On the other hand, iron deficiency is also harmful to cells because iron functions as a cofactor in many enzymatic reactions [6,7,8]. The regulation of iron metabolism occurs via the high-affinity system represented by the Fet3/Ftr1 complex, which includes ferroxidase and iron permease, in *S. cerevisiae* [9]. The low-affinity iron uptake system represented by Fet4 also has an important role in the regulation of iron metabolism [10]. Fet3 engages copper as a cofactor, and Fet3 does not perform normal functions without copper, as reported previously [11]. Therefore, copper supplementation is an important factor for maintaining iron homeostasis, and several genes involved in copper homeostasis are regulated by iron at the transcriptional level [11,12]. Therefore, iron and copper are regulated in a mutually dependent manner in *S. cerevisiae*.

Copper uptake is regulated strictly, similar to iron metabolism, because of the toxic effect of copper overloading [13]. In *S. cerevisiae*, copper is taken up from the environment by two copper transporters, namely Ctr1 and Ctr3, which are localized at the plasma membrane [14], and copper taken up by the transporters is delivered to target organelles by intracellular delivery systems, such as those initiated by Atx1, Ccs, and Cox17, for copper delivery to the Golgi, the Sod1 protein, and the mitochondria, respectively [15,16]. The expression of genes that encode copper transporters is regulated by Mac1 transcription activators at the transcriptional level [17]. Mac1 has a copper-binding domain and a DNA-binding domain and senses cellular copper concentration [18]. Mac1 homologs have been found in other fungal species, for example, AfMac1 and CaMac1 from *Aspergillus fumigatus* and *Candida albicans,* respectively [19,20]. Interestingly, AfMac1 has a dual transcriptional activator that regulates copper and iron metabolism [19]. This report provides direct evidence that copper and iron metabolism are very closely connected, as identified in *S. cerevisiae*.

Iron metabolism is also affected by nutrients, and protein kinase A (PKA) has already been reported as a key regulator of iron homeostasis in *S. cerevisiae*. PKA regulates cell growth in response to carbon source utilization and is activated by cAMP in response to glucose concentration [21]. PKA is a heterotetramer composed of two Bcy1 and two catalytic subunits encoded by the Tpk genes (Tpk1, Tpk2, and Tpk3) [22]. It has been suggested that Bcy1 is involved in the inhibition of ferrireductase activity of Fet3 induced by iron depletion. Tpk2p is required for transcriptional regulation of the genes involved in the high-affinity iron uptake pathway [23]. Furthermore, the iron permease Ftr1 was identified as being involved in PKA activation [24]. These reports indicate that sugar utilization regulates the high-affinity iron uptake pathway.

However, the involvement of nitrogen sources in metal homeostasis has not yet been reported. Nitrogen sources are also macronutrients required by living organisms, and their utilization is regulated by diverse regulatory systems. In this report, we describe the effect of nitrogen sources on copper homeostasis in *S. cerevisiae*.

## 2. Materials and Methods

### 2.1. Yeast Strains, Media, and Culture Conditions

The yeast strains used in this study were *S. cerevisiae* wild-type BY4741 (*MAT a*, *his3Δ1, leu2Δ0, met15Δ0,* and *ura3Δ0*) and YPH499 (*MAT a, ura3-52, leu2-Δ1, lys2-801, his-Δ200, trp1-Δ63, ade2-101*). The cells were grown to mid-log phase in YPD or synthetic defined (SD) medium in this study. *Δmac1, Δgpr1, Δgpa2, Δmep2, Δras1, Δras2, Δtpk1, Δtpk2, Δtpk3, Δste11,* and *Δcph1* deletion strains (Takara Bio USA Co., San Jose, CA, USA) and *FET3-HA* were derived from the BY4741 strain. *CTR1-HA* was derived from the YPH499 strain. Seed cultures were performed at 30 °C with shaking at 200 rpm overnight. The yeast strains were grown in SD medium (0.17% yeast nitrogen base without amino acids and ammonium sulfate, 0.5% NH4(SO4)2, 2% glucose) supplemented with the necessary auxotrophy-related components, which were purchased from Difco (BD Difco Co., Franklin Lakes, NJ, USA). Copper-limited media were prepared by adding 100 μM bathocuproinedisulfonic acid (BCS). All cultures were grown at 30 °C.

### 2.2. Plasmid Construction

The *PGK1* promoter region was amplified by PCR using the appropriate primer sets (Table S1) and introduced into pRS316, and the resulting plasmid was named 316-*PGK1*. *MAC1* without a stop codon, the *HA* epitope (hemagglutinin), and the *ADH1* terminator were amplified by PCR using the appropriate primer sets (Table S1) from yeast genomic DNA and cloned into pFA6a-3HA-kanMX6. The *HA* epitope and *ADH1* terminator were introduced into the 316-P_PGK1_-MAC1 plasmid without a stop codon, and the plasmid was transformed into the *Δmac1* strain to exclude endogenous Mac1 activity.

### 2.3. Nitrogen Starvation

For nitrogen starvation, cells cultured in YPD medium were washed with 0.9% NaCl solution three times, transferred to SD-N medium (0.17% yeast nitrogen base without amino acids and ammonium sulfate, 2% glucose) at an OD600 of 1.0, and incubated at 30 °C with shaking at 200 rpm for the indicated times. BCS or the indicated concentration of copper was added to the SD-N medium when necessary.

### 2.4. Northern Blot Assay

Total RNA was extracted from yeast cells using glass beads for cell lysis (Sigma-Aldrich) and purified using RNAiso Plus (Takara Co., Kusatsu, Shiga, Japan). The total RNA was resolved on a 1% agarose gel containing formaldehyde. A nylon membrane (Hybond-N+, GE Healthcare, Chicago, IL, USA) was used to transfer RNA from the gel. To make the probe DNA, the corresponding DNA fragments were amplified from the coding regions of the indicated genes by PCR using the indicated primer sets (Table S1). Labeling of radioisotopes of DNA probes was performed using a random priming kit (GE Healthcare, Chicago, IL, USA). Hybridization was performed at 65 °C overnight. *ACT1* and rRNA were used as loading controls.

### 2.5. Iron Uptake Assay

Briefly, the cells (WT and indicated deletion mutants) were grown to mid-log phase in the indicated media. The cells were washed three times with citrate buffer (50 mM sodium citrate and 5% glucose; pH 6.5). ^55^FeCl2 (1 μM, PerkinElmer) was reduced with ascorbic acid (Sigma-Aldrich) for ferrous iron uptake but not for ferric iron uptake. The cells were incubated with ^55^FeCl_2_ for 1 h at 30 °C for the samples and at 4 °C for the negative control. After incubation, the cells were washed five times, and the radioactivity was measured in triplicate using a liquid scintillation counter (Beckman Coulter, Brea, CA, USA). All iron uptake assays were performed independently at least three times.

### 2.6. Western Blot Assay

Total protein samples were prepared from the cells using acid-washed glass beads in lysis buffer containing 50 mM Tris–HCl (pH 7.5), 100 mM NaCl, 1 mM EDTA, dithiothreitol and protease inhibitor cocktail (Complete Mini, Roche Applied Science, Penzberg, BA, Germany), and 1% Triton X-100. Protein concentrations were measured using a BCA kit (Pierce, Appleton, WI, USA) according to the manufacturer’s protocol. Cell lysates were resolved by SDS-PAGE, and the gels were transferred to nitrocellulose membranes (Amersham, Bucks, England). Then, the membranes were blocked with 5% skim milk in TBST (25 mM Tris–HCl (pH 8.0), 150 mM NaCl, and 0.05% Tween 20) before hybridizing with the anti-HA antibody (Santa Cruz Biotechnology, Dallas, TX, USA). PGK1 was used as a loading control.

### 2.7. Statistical Analysis

All experiments, including iron uptake assay and western blotting, were performed in triplicate or duplicate respectively. The differences among the groups were assessed using Student’s *t*-test for unpaired samples, and *p*-values less than 0.05 were considered significant. Statistical significance was marked with asterisks. One asterisk (*) indicates that the *p*-value is less than 0.05, and two asterisks (**) indicate a *p*-value less than 0.01.

## 3. Results

### 3.1. Deletion of the Genes Involved in Glucose and Nitrogen Metabolism Resulted in a Decrease in Iron Uptake

**Figure 1:**
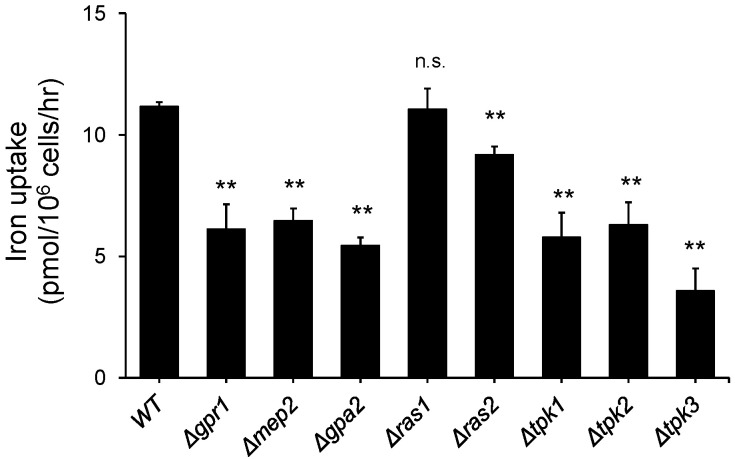
Deletion of the genes involved in glucose and nitrogen metabolism resulted in a decrease in iron uptake. Each indicated deletion mutant of *S. cerevisiae* was cultured in YPD medium until mid-log phase, and an iron uptake assay was performed as described in the Materials and Methods section. The assay was performed in triplicate. n.s., not significant; **: *p* < 0.01 indicates a significant difference compared to wild type.

**Figure 2:**
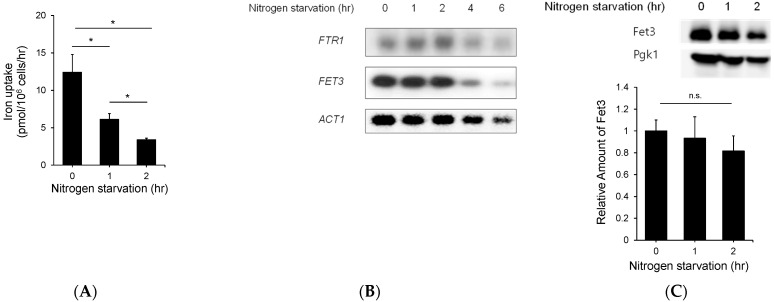
The expression of genes involved in high-affinity iron uptake is not affected by nitrogen starvation. (**A**) The wild-type strain of *S. cerevisiae* was cultured in YPD medium, and nitrogen starvation was performed as described in the Materials and Methods section for the indicated times. In addition, an iron uptake assay was performed. (**B**) The expression of the genes involved in the high-affinity iron uptake system, namely FTR1 and FET3, was investigated. The wild-type cells were starved of nitrogen for the indicated times, total RNA was extracted, and then northern blotting was performed for the indicated genes. (**C**) Additionally, western blotting was performed to confirm the protein level of Fet3 under nitrogen starvation. The wild-type cells were starved of nitrogen for the indicated times, total proteins were extracted, and then, western blotting was performed for Fet3. The expression of the protein was showed quantitatively. n.s., not significant; *: *p* < 0.05 indicates a significant difference compared to 0 nitrogen starvation time.

To investigate the role of carbon and nitrogen sources in iron metabolism, we measured the iron uptake activity of the selected mutants with deletions of genes involved in carbohydrate and nitrogen metabolism. Gpr1, Gpa2, Tpk1, Tpk2, and Tpk3 are known as proteins involved in the glucose signaling pathway in *S. cerevisiae* [22]. Mep2 is known as an ammonia transporter and has a major role in nitrogen metabolism in *S. cerevisiae* [25]. Furthermore, Ras1 and Ras2 are known to take part in carbohydrate and nitrogen metabolism [26]. As shown in Figure 1, the indicated deletion mutant was cultured in YPD medium until mid-log phase, and iron uptake activity was measured as described in the Materials and methods section. As reported previously, some deletion mutants that were defective in carbohydrate metabolism showed lower iron uptake activity than the wild type. Interestingly, the Mep2 deletion mutant showed lower iron uptake activity than wild-type cells. To understand the effect of ammonia utilization on iron uptake, the cells were subjected to nitrogen starvation for 1 or 2 h, as described in the Materials and Methods section. As shown in Figure 2A, the iron uptake activity of the cells cultured in nitrogen starvation medium was decreased compared with that of the cells cultured in standard SD medium and decreased further upon starvation for 2 h. This result implies that ammonia utilization is involved in iron metabolism. To further investigate how nitrogen utilization affects iron uptake, we performed northern blot analysis with *FET3* and *FTR1,* which encode major high-affinity membrane iron transporters. As shown in Figure 2B, the cells were starved for nitrogen for the indicated times, and total RNA was extracted from the cells. The expression of *FTR1* and *FET3* at the transcriptional level was not changed until 2 h after nitrogen starvation and began to decrease 3 h after nitrogen starvation, although iron uptake activity decreased dramatically 2 h after nitrogen starvation. These results showed discrepancies between iron uptake and the northern blot data. Furthermore, we analyzed the Fet3 level to determine whether nitrogen starvation affects the translational level of the reductive iron uptake pathway. As shown in Figure 2C, the cells were cultured in nitrogen starvation medium for 1 or 2 h, and then, total proteins were extracted to perform western blot analysis. The Fet3 level was measured and was found to not have decreased as much as the iron uptake activity. These results indicate that nitrogen starvation does not directly affect Fet3/Ftr1.

### 3.2. Nitrogen Starvation Downregulated the Expression of Genes Involved in Copper Metabolism

**Figure 3:**
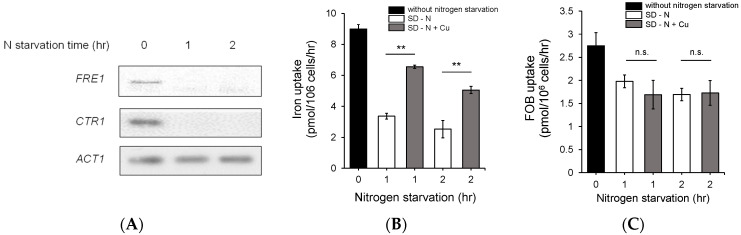
Nitrogen starvation downregulated the expression of the genes involved in copper metabolism. (**A**) Expression of the genes involved in copper uptake was investigated to determine whether gene expression is regulated by nitrogen starvation. The wild-type cells were cultured and starved of nitrogen as described in the Materials and Methods section, and total RNA was extracted. ACT1 was used as a loading control. (**B**) The effect of copper on iron uptake under nitrogen starvation conditions was investigated. The wild-type cells were starved of nitrogen with (white column) or without (gray column) copper, and then, an iron uptake assay was performed. (**C**) A ferrioxamine B (FOB) uptake assay was performed to identify the effect of copper on FOB uptake. Desferol and Fe^55^ were mixed at equal concentrations prior to the uptake assay, and then, the uptake assay was performed as described for the free iron uptake assay. n.s., not significant; **: *p* < 0.01 indicates a significant difference compared to other groups.

Reductive iron uptake is regulated by copper utilization because copper is a cofactor of the ferroxidase Fet3, which is a component of the reductive iron uptake system [11]. To understand how nitrogen starvation downregulates iron uptake, we investigated the effect of nitrogen utilization on copper metabolism to determine whether copper deficiency was induced by nitrogen starvation. As shown in Figure 3, the cells were cultured in nitrogen starvation medium for the indicated times, and northern blot analysis was performed for *FRE1* and *CTR1,* which encode a membrane ferric reductase and high-affinity copper transporter, respectively, and have important roles in copper uptake in *S. cerevisiae*. As shown in Figure 3A, the gene expression of *FRE1* and *CTR1* was dramatically downregulated by nitrogen starvation, and no transcripts were detected after nitrogen starvation for 1 h. This result indicates that nitrogen starvation downregulates the gene expression of *CTR1* and *FRE1*. To further investigate the effect of copper on nitrogen starvation, we added copper to nitrogen starvation culture medium and measured the iron uptake activity. As shown in Figure 3B, the cells were cultured in nitrogen starvation medium, and copper was added to the medium directly at a final concentration of 20 μM during nitrogen starvation. Interestingly, iron uptake activity was partially recovered by the addition of copper to the SD medium in which the cells were cultured, and these results provide clues regarding how nitrogen starvation inhibits iron uptake.

To investigate whether nitrogen starvation inhibits siderophore uptake, we measured ferrioxamine B (FOB) uptake after nitrogen starvation. FOB uptake activity was decreased less than the free iron level by nitrogen starvation, which is explained by FOB uptake using different machinery from the reductive iron uptake system [27]. Copper supplementation did not restore FOB uptake activity, as shown in Figure 3C. These results indicate that nitrogen starvation specifically affects the reductive iron uptake system.

### 3.3. Nitrogen Starvation Downregulated Mac1 Expression at the Translational Level

**Figure 4:**
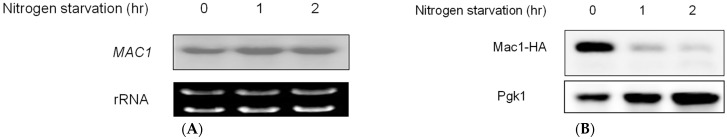
Nitrogen starvation downregulated Mac1 expression at the translational level. (**A**) To identify the reason why the expression of the genes involved in copper uptake was downregulated, northern blotting was performed for MAC1, which is a copper-responsive transcription activator. The cells were starved of nitrogen for the indicated times, and total RNA was extracted. (**B**) To confirm whether nitrogen starvation affects Mac1 expression at the protein level, western blotting was performed. The Mac1-HA strain, which harbors an HA-tagged version of Mac1 in the genome, was cultured and starved of nitrogen for the indicated times, and total proteins were extracted. Pgk1 was used as a loading control.

**Figure 5:**
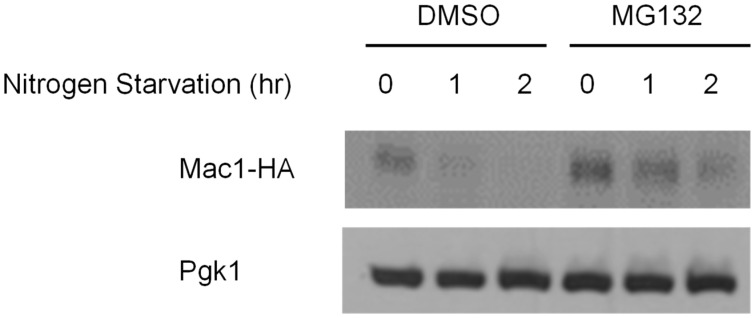
Mac1 is regulated by proteasome-dependent protein degradation under nitrogen starvation. To identify the mechanism of Mac1 regulation by nitrogen starvation, MG132, which is a proteasome inhibitor, was used. The cells were starved of nitrogen with or without MG132 in the medium, total proteins were extracted, and then, western blotting was performed. PGK1 was used as a loading control.

As shown in Figure 3, we found that the expression of *FRE1* and *CTR1* was downregulated by nitrogen starvation, but the underlying mechanisms was unknown. One possibility was that Mac1 may be regulated by nitrogen starvation. Mac1 is a copper-responsive transcriptional activator of *S. cerevisiae*. Mac1 regulates the expression of genes involved in copper metabolism, and Mac1 homologous transcriptional activators have been identified in many fungal species. To investigate the effect of nitrogen starvation on Mac1, we performed northern blot analysis to identify the expression of *MAC1* at the transcriptional level. As shown in Figure 4A, yeast cells were cultured in SD medium and then starved of nitrogen for 1 and 2 h, followed by total RNA extraction. Interestingly, the expression of *MAC1* was not decreased by nitrogen starvation, and the same amount of transcript was found in nitrogen-starved cells. We investigated the expression of Mac1 at the protein level and found that the protein level decreased dramatically after nitrogen starvation for 1 h, as shown in Figure 4B. These results indicate that nitrogen starvation downregulates Mac1 expression at the protein level and then downregulates the genes involved in copper metabolism. Next, we attempted to identify the regulatory mechanism of Mac1 by nitrogen starvation. Many regulatory mechanisms have been reported at the protein level, and proteasome-dependent and protease-dependent regulatory mechanisms have been reported [28,29]. We first investigated the proteasome-dependent pathway and treated cells in starvation medium with MG132, which is an inhibitor of proteasome-dependent protein degradation [30]. As shown in Figure 5, the cells were cultured in SD medium, and then, nitrogen starvation was performed for 1 or 2 h with the addition of MG132 to the medium. Interestingly, nitrogen starvation dramatically downregulated Mac1 in DMSO-treated cells as a negative control. However, treatment with MG132 slowed protein degradation compared with DMSO treatment, as shown in Figure 5. These results indicate that nitrogen starvation downregulates Mac1 expression at the protein level in a proteasome-dependent manner and then downregulates the genes involved in copper metabolism.

## 4. Discussion

In this study, we investigated the role of nitrogen utilization in iron metabolism. *S. cerevisiae* uses nitrogen sources selectively. In the absence of a preferred nitrogen source or at low nitrogen concentrations, yeast absorbs nitrogen by positive regulation of the ammonium permease Mep1-3 by the Npr1 kinase activated through TORC1 [31]. Under nitrogen starvation, cells induce autophagy by producing autophagosomes to switch the cell cycle to the G0 phase [32]. Cells adopt this strategy to survive nutrient deficiencies, and this method allows cells to stabilize the amino acid pool and maintain protein synthesis. Autophagy is induced by sensing the depletion of various nutrients, and nitrogen is known as the factor that most rapidly induces autophagy, and autophagy is induced after 4 h from the nitrogen starvation [33].

Here, we observed that iron uptake was partially inhibited in *S. cerevisiae* with mutation of mep2, a membrane ammonium transporter. This result implies that the deletion of mep2, which functions downstream of TOR signaling, resulted in a decrease in iron uptake activity. However, the high-affinity iron ferroxidase Fet3 or high-affinity iron permease Ftr1 did not show transcriptional or translational changes under nitrogen starvation for 2 h (Figure 2). These results suggest that the iron uptake machinery is not directly regulated by nitrogen starvation. On the other hand, we found that nitrogen starvation reduced the expression of Mac1, a copper response transcription factor, at the post-translational level but not at the transcriptional level. Mac1 controls the activity of copper regulons by directly sensing copper through the copper fist structure. We also found a decrease in Ctr1 expression after nitrogen starvation (Figure 3). Fet3 is a copper-containing oxidase whose activity is regulated by the presence of copper. As a result, inhibition of iron uptake by nitrogen deficiency was partially restored when copper was added to the cells (Figure 3B). This confirmed that the reduction in iron uptake under nitrogen starvation was caused by copper depletion. This result also indicates that inhibition of iron uptake by nitrogen starvation is not caused by autophagy because autophagy is induced after 4 h from nitrogen starvation, as described earlier [33].

It remains unknown how nitrogen starvation regulates Mac1 at the translational level. The detailed regulatory mechanism of Mac1 at the protein level has not been reported even though it has been reported that Mac1 is regulated by proteases under high-copper conditions [34]. Our data showed that Mac1 is regulated in a proteasome-dependent manner because MG132 partially inhibited the protein degradation caused by nitrogen starvation (Figure 5). This result is a new observation of proteasome-dependent Mac1 degradation. Therefore, we can propose a model for the mechanism of nitrogen starvation-mediated regulation of Mac1. Nitrogen starvation activates proteasome-dependent protein degradation in an unidentified manner, and Mac1 is the target. To understand the detailed regulatory mechanism, further research is needed.

Trace elements, such as iron and copper, are important nutrients for cell growth and division, and in particular, metal ions are essential as cofactors of numerous enzymes [35,36,37]. Recently, it has been reported that transceptors of metals, such as Ftr1 and Zap1, activate PKA pathway in *S. cerevisiae,* and these transceptors participate in metal mediated carbohydrate metabolism [24]. Metal homeostasis, which must be tightly regulated, is even more important when cells are under nutrient depletion. This is because transition metals can cause excessive or deficient stress to cells. However, even if iron cannot be imported from outside the cell, the cell can use the iron preserved in vacuoles as a transition metal for minimal biological activity. Therefore, inhibition of iron absorption is more appropriate for the survival of cells in nitrogen-depleted conditions.

In this study, we were able to elucidate the mechanisms by which yeast cells regulate the metabolic activity of copper and iron through Mac1 degradation when subjected to nitrogen starvation. In addition, it was confirmed that the mechanisms underlying the tight regulation of copper and iron influence each other even under extreme stress.

## Data Availability

Not applicable.

## Supplementary Materials

The following are available online at https://www.mdpi.com/article/10.3390/jof7090756/s1, Table S1: Primers used in this study.
