# Supplementary material for: Regulation of Copper Metabolism by Nitrogen Utilization in Saccharomyces cerevisiae
Source: J Fungi (Basel). 2021 Sep 14;7(9):756. doi: 10.3390/jof7090756 (PMC8469692; doi:10.3390/jof7090756)
Supplement: Supplementary file 1 [file jof-07-00756-s001.zip › jof-1356198-supplementary.pdf]

Table S1. Primers used in this study.

No.	Primer name	Sequence (5′ → 3′)
1	*FTR1*_north_F	AGGCAATCGGGGAACATGAC
2	*FTR1*_north_R	CCAATCTCCACCAACGACCG
3	*FET3*_north_F	CCTTCTTGACGCAATGTCCAAT
4	*FET3*_north_R	TACGTCGTATCGGGCTGGA
5	*ACT1*_north_F	ACTGAAGCTCCAATGAACCCT
6	*ACT1*_north_R	CAGCAGTGGTGGAGAAAGAGT
7	*FRE1*_north_F	GCTCATGGCGAACGAGACAG
8	*FRE1*_north_R	GACCCTTGCCTCGAGTTGTA
9	*CTR1*_north_F	CAGTAGCATCGAGTATGGCGT
10	*CTR1*_north_R	TCCATCCCTGAAGAGCTACTGT
11	*MAC1*_north_F	TGCACAAATTGAAGGTGACTGC
12	*MAC1*_north_R	GGCTTCGCTCTTTCTTCTCAC
13	T7	TAATACGACTCACTATAGGG
14	*MAC1*_del_stop_*Xho*1_R	TCCTCGAGTGAAGTGGTGGCATCGCTTAG
15	3HA_*Xho*1_R	GATCCTCGAGCGGATCCCCGGCTTAATTAACA
16	3HA_*Kpn*1_R	GATCGGTACCCCGGTAGAGGTGTGGTCAATAA
